# Characterization of strain degeneration in the RL-P37 strain lineage of *Trichoderma reesei*

**DOI:** 10.1186/s13068-025-02729-z

**Published:** 2026-01-06

**Authors:** Caroline Danner, Armin Gabriel, Christian Zimmermann, Robert L. Mach, Yuriy Karpenko, Igor Nikolaev, Sharief Barends, Astrid R. Mach-Aigner

**Affiliations:** 1 Christian Doppler Laboratory for Optimized Expression of Carbohydrate‐Active Enzymes, Institute of Chemical, Environmental and Bioscience Engineering, TU Wien, Gumpendorfer Str. 1a, 1060 Vienna, Austria; 2 Institute of Chemical, Environmental and Bioscience Engineering, TU Wien, Gumpendorfer Str. 1a, 1060 Vienna, Austria; 3 IFF Health & Biosciences, Willem Einthovenstraat 4, 2342BH Oegstgeest, Netherlands

**Keywords:** Cellulase, *Trichoderma reesei*, Strain degeneration, Morphology

## Abstract

Spontaneous strain degeneration, defined as the loss of an essential biological function during prolonged usage, is frequently observed in microorganisms and poses a significant challenge to the biotechnology industry. In *Trichoderma reesei*, a filamentous fungus widely used for large-scale cellulase production, spontaneous loss of cellulase productivity has been reported. However, studies on this phenomenon have focused solely on industrial strains derived from the Rut-C30 lineage. This study analyzes strain degeneration in a different industrial lineage of *T. reesei*, RL-P37, and its hypercellulase-producing descendant, GEN-3A. We found that RL-P37 and GEN-3A are also affected by the degeneration phenomenon, with the highly productive GEN-3A showing greater susceptibility. The degenerated phenotype was characterized by reduced cellulase productivity, altered growth behavior, and distinct morphological changes. In particular, cellulase hyperproduction was associated with bulbous, highly branched hyphae, while these morphological traits were lost in degenerated isolates. Our study establishes a framework for characterizing strain degeneration in *T. reesei*, highlights the trade-off between productivity and stability, and identifies distinctive morphological signatures linked to cellulase hyperproduction and degeneration, which may serve as early phenotypic indicators for industrial strain monitoring.

## Introduction

The filamentous fungus *Trichoderma reesei* is applied for the large-scale production of carbohydrate-active enzymes, such as cellulases or xylanases. Cellulase and xylanases are among the most widely used enzymes in industry. Applications range, for example, from pulp and paper, food and textile industry, to bioethanol production [1].

**Fig. 1:**
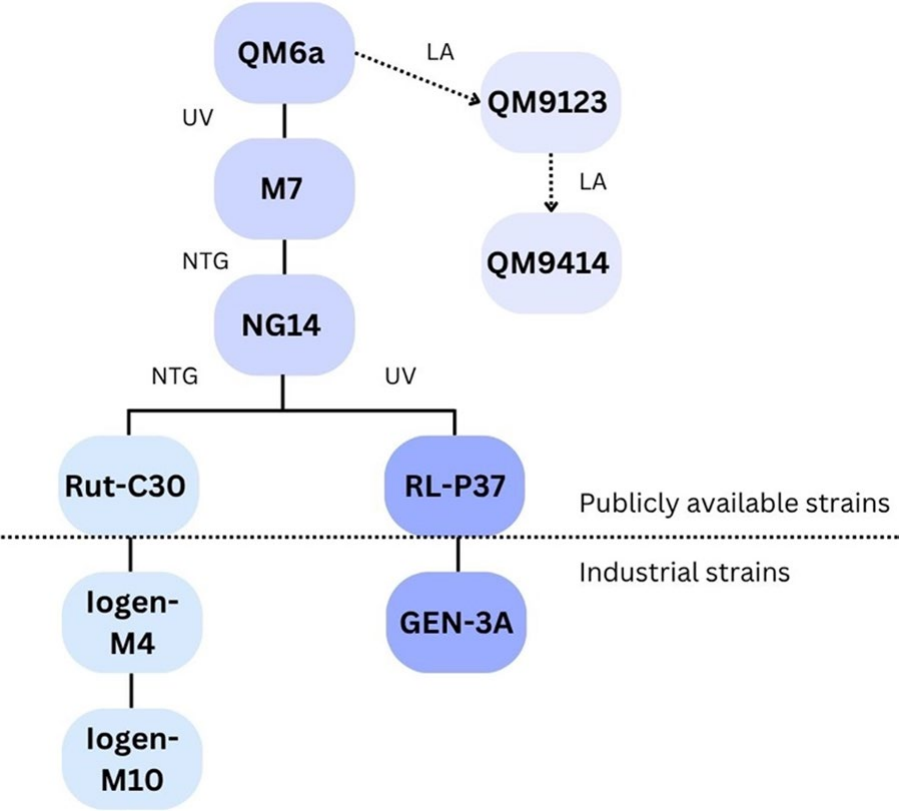
Pedigree of *T. reesei* strains. Strain names and mutagenesis methods are given. LA, linear acceleration; UV, UV-light; NTG, nitrosoguanidine

All hypercellulase-producing strains of *T. reesei* derive from the wild-type isolate QM6a from the Solomon Islands [2]. Two main lineages of hypercellulase-producing strains were developed by successive rounds of classical mutagenesis, with one of them again being developed in two sub-lineages: from NG14, the lineage diverges into two branches, leading to Rut-C30 and RL-P37. Rut-C30, which has 15 to 20 times the enzyme activity of QM6a, was derived from NG14 by another round of UV-mutagenesis and selection for release of carbon catabolite repression (CCR) [3, 4]. Among numerous other mutations, the *cre1* gene is truncated in Rut-C30, partially releasing CCR under glucose conditions [5]. RL-P37 is also derived from NG14 by subjecting it to UV-mutagenesis, but it is still under CCR when glucose is available [6]. Rut-C30 and RL-P37 are the best cellulase producers that are publicly available and are frequently used in research. Of course, even higher cellulase-producing strains were derived from them for industrial production [7]. A pedigree of the different *T. reesei* strains relevant for cellulase production can be found in Fig. 1.

While hyperproduction of cellulases is a highly desirable trait for industrial applications, it comes at a cost. It has been reported that hyperproductive *T. reesei* strains spontaneously lose cellulase productivity during the industrial production process. This so-called phenomenon of strain degeneration was described in QM6a and Rut-C30 and its industrial descendants, Iogen-M4 and Iogen-M10 [8]. A protocol for induced strain degeneration (ISD) was developed to reproducibly study the degeneration in small-scale in the lab. This protocol allows for quantification and isolation of the non-producing part of the population referred to as (cel–) and the still cellulase-producing part, namely (cel +) [8]. However, the phenotypic and morphological characteristics of these (cel–) and (cel +) isolates have not been previously described in detail.

Degeneration was found to correlate with the productivity of the strain. The more productive the strain, the more susceptible it is to losing productivity. The wild-type QM6a hardly degenerates (0–10%), while the highest producer, Iogen-M10, lost its production capacity almost entirely (90–100%) when exposed to the ISD protocol. The underlying mechanisms leading to this have not yet been fully deciphered, but it is suggested that epigenetic mechanisms cause it. In particular, DNA methylation was recently shown to impact the degeneration phenomenon and cellulase production in general [9].

Similar phenomena of spontaneous production losses are reported in other biotechnologically applied filamentous fungi, such as *Aspergillus* and *Penicillium* (reviewed in [10]). The mechanisms driving these declines vary and can be attributed to genetic or epigenetic factors, as well as extrinsic influences such as culture conditions, aging, or stressors [10]. Despite the prevalence of these issues, there is a significant gap in understanding their underlying causes, highlighting the need for further research aiming to overcome these production declines.

Fungal morphology is a key factor for efficient product formation and secretion in industrial production processes [11–13]. Many studies have been conducted to alter the morphology of *T. reesei* to improve cellulase production. It was observed that shorter, highly branched hyphae are associated with optimal cellulase production [14–17]. In industrial settings, both pellet-like and dispersed filamentous growth are currently applied for cellulase production [18].

Moreover, fungal degeneration often correlates with distinct morphology changes, enabling differentiation between the non-producing and the still-producing subpopulations of a culture [10]. For instance [19], the decline in penicillin biosynthesis in *Penicillium chrysogenum* is associated with various morphological alterations, including the formation of fluffy-white sectors in the mycelium, diminished spore pigmentation, and an overall reduction in conidiation [20–22]. In the case of degenerated *T. reesei* (cel–) cells, a shift from a more bulbous to a more elongated cell structure is described. However, these changes were not shown in the cited study [8]. This suggests that further investigation into the morphological characteristics of the degenerated *T. reesei* phenotypes could be helpful for (early) monitoring any loss in cellulase production.

This study aimed to find out whether strain degeneration has a broader impact for industrial *T. reesei* strains besides the lineage from Rut-C30. It also offers an in-depth characterization of the phenomenon. Specifically, we investigated whether strain degeneration occurs in strains derived from the RL-P37 lineage and, if so, whether its severity is linked to the productivity level of individual strains. To address these questions, we adapted the earlier described protocol for ISD for RL-P37 and its hypercellulolytic descendant GEN-3A and compared their degeneration behavior with that of the Rut-C30 lineage. In addition to quantifying degeneration rates, we characterized (cel +) and (cel-) phenotypes concerning cellulase activity, growth, and morphology. We identified distinct morphological characteristics in the hyperproductive strain that were diminished in the degenerated variant. Potentially, this could serve as a phenotype-based marker for early detection and screening of strain degeneration. The understanding gained in this study serves as the basis for future experiments concerning the exact mechanism underlying the strain degeneration.

## Materials and methods

### Strains and cultivation conditions

The following *T. reesei* strains were used for this study: the wild-type strain QM6a (ATCC 13631), the strain Rut-C30 (ATCC 56765), which is described as hypercellulytic and was derived by two rounds of mutagenesis and screening from QM6a [2, 3]. The *T. reesei* strain RL-P37 and its hypercellulytic descendant GEN-3A were provided by IFF Nutrition & Biosciences (Oegstgeest, Netherlands). The strains were maintained on potato dextrose agar (PDA) plates. For short-term storage, the strains were kept at 4 °C as whole plate cultures and spore solutions, and for long-term storage as spore suspensions in 25% glycerol at − 80 °C.

Cultivation for enzyme production was performed in 100-mL shake flasks using 20 mL of Mandels–Andreotti (MA) minimal medium [23] containing 1% (w/v) lactose. The concentration of the inoculum was 10^6^ spores/mL. The strains QM6a and Rut-C30 were cultivated at 30 °C and 180 rpm for 48 h. The first cultivation of RL-P37 and GEN3A for cellulase activity determination was performed as described above for QM6a and Rut-C30. For subsequent experiments, the medium was changed to production medium (i.e., a minimal medium with cellulase-inducing compounds, provided by IFF), and cultivation was at 25 °C and 160 rpm for 96 h. After the cultivation, filtration through a miracloth filter separated the mycelium from the supernatant.

### Induced strain degeneration of T. reesei

Strain degeneration was initially determined using the ISD protocol established for the *T. reesei* Rut-C30 lineage [8]. RL-P37, GEN-3A, QM6a, and Rut-C30 were subjected to two rounds of the ISD with and without adding 20 mM dithiothreitol (DTT). The protocol was performed as described, with the only difference that the carboxymethylcellulose (CMC) agar plates were stained for 3 to 5 min with Gram’s Iodine solution (Sigma Aldrich, St. Louis, US) instead of Congo red [24]. Later, the protocol was adapted to the RL-P37 lineage and is described in detail in the results part 3.2. From the adapted protocol (cel-) and (cel +) strains were isolated for further investigation. Degeneration rates were calculated as described by Martzy et al. [8]. The degeneration rate represents the percentage of non-cellulase producing colonies (cel-) within the total population composed of both, cellulase producing (cel +) and non-producing (cel-) colonies [8].

### Determination of the homogeneity of spore populations

Spore solutions of the *T. reesei* strains Rut-C30, RL-P37, and GEN-3A and the respective (cel-)/(cel +) isolates were diluted and spread out on CMC agar plates in biological duplicates to achieve around 30 to 50 colonies. The percentage of (cel-) colonies in the total population was calculated from the whole spore poulation, accordingly to the degeneration rate described by Martzy et al. [8].

### Cellulase activity and protein content

The cellulase activity in the supernatants was measured using the azo-carboxymethylcellulose assay (Megazyme, Wicklow, Ireland), and the protein content was determined using the Bradford assay (Bio-Rad protein assay, Hercules, California, USA) following the manufacturer’s guidelines. The biomass was quantified by drying the mycelia at 80 °C overnight and measuring the dry weight. Strains were grown in biological triplicates, and cellulase activity and protein content were measured in technical triplicates if not stated otherwise.

Eventual changes in cellulase activity of the *T. reesei* strains and their respective (cel-)/(cel +) isolates was monitored by serial sub-cultivation on MA medium agar plates containing 0.5% CMC (MA CMC) in biological triplicates. For this, (cel-) and (cel +) colonies were cut out with a 4 mm metal cylinder and put on fresh MA CMC agar plates supplementedwith 0.1% Igepal CA-630 (octylphenoxypolyethoxyethanol). A 4-mm agar plug of the non-degenerated strains pregrown on PDA was used as a control. The MA CMC agar plates were incubated for 48 h at 30 °C, then the colony size was recorded by measuring the diameter. A new 4-mm agar plug was transferred to a new MA CMC agar plate, then the plates were kept for 5 h at 50 °C and stained with Gram’s iodine solution to measure the halo size [24]. The colony and halo sizes were recorded over a total time of 432 h. The image processing application Fiji/ImageJ 1.54 g Java 1.8.0_345 (64-bit) was used to measure colony and halo size.

### Nanolive 3D cell-explorer imaging of T. reesei

The sample preparation method described in Fritsche et al. [25] was optimized for *T. reesei.* A number of 9.1 × 10^7^ spores of the *T. reesei* strains GEN3A, GEN3A (cel-), and GEN3A (cel +) were inoculated in 500 µL cellulase production medium (provided by IFF) or on minimal medium with glucose in an Ibidi µ-dish (35 mm). After 3 h of settling, it was washed 2 times with 500 μL of fresh medium and filled with 1.5 mL of the respective medium. The 3D Cell Explorer-fluo microscope (Nanolive SA, Ecublens, Switzerland) was used for 3D holotomographic images and 4D time-lapse imaging*.* For time-lapse imaging, the cultivation dish was placed in a stage-top incubator system, which was maintained at 30 °C with a temperature controller (Okolab, Naples, Italy) and protected from dehydration by placing water reservoirs into the incubation chamber. Single acquisitions (3D) were performed after 18 h incubation in the same set-up. The image stacks of the 3D and 4D acquisitions were processed as described in Fritsche et al. [25], using the image processing application Fiji/ImageJ 1.54 g Java 1.8.0_345 (64-bit).

## Results and discussion

### *Strain degeneration also affects the RL-P37 lineage of* T. reesei

**Fig. 2:**
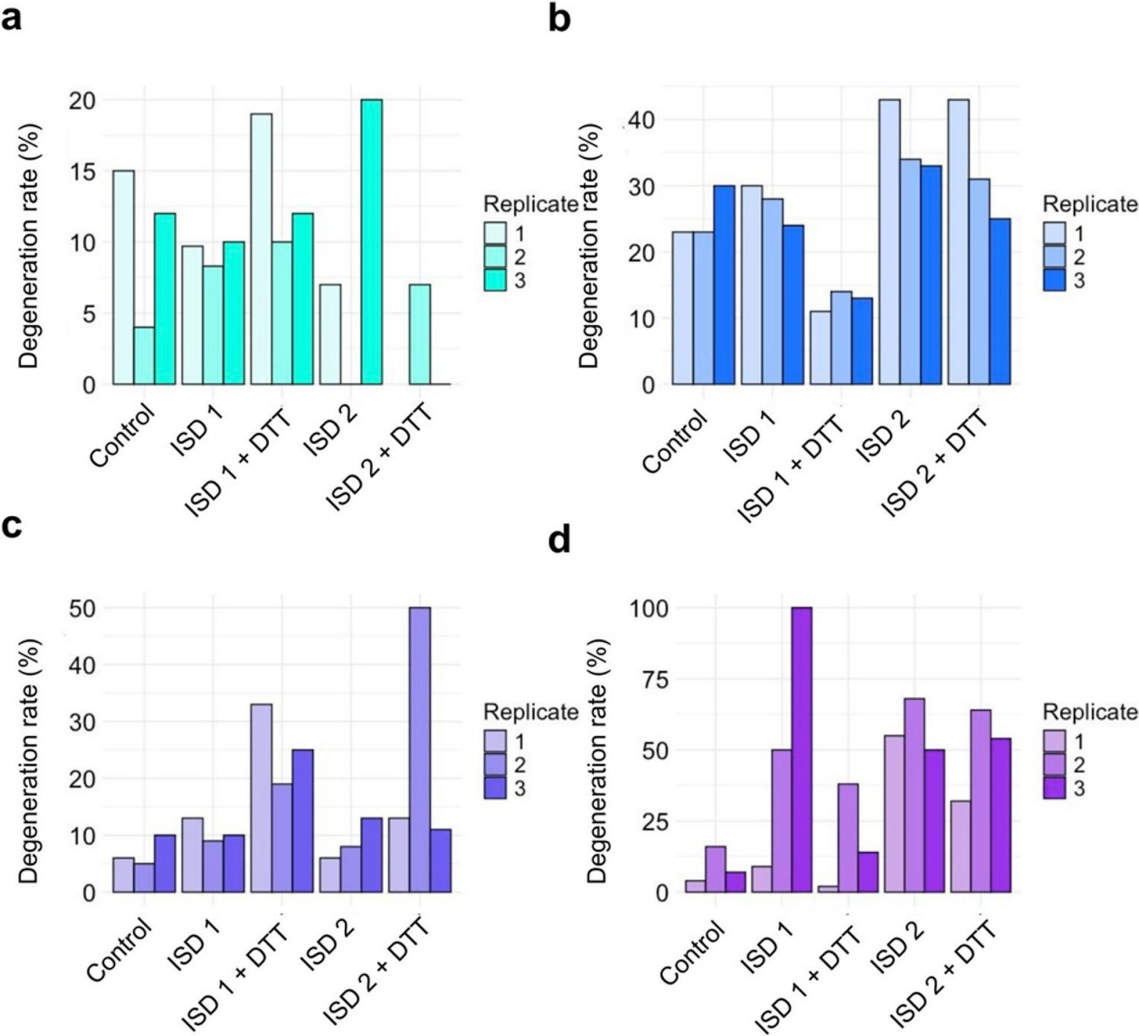
The (cel-) occurrence after artificially induced strain degeneration in different *T. reesei* strains. The strains QM6a (**a**), Rut-C30 (**b**), RL-P37 (**c**), and GEN-3A (**d**) were subjected to the induced strain degeneration (ISD) protocol in two rounds (1, 2) without and with the addition of 20 mM dithiothreitol (DTT). The degeneration rate is the occurrence of the (cel-) population before (control) and after the ISD protocol and is given as a percentage of the total population. Values of the three biological replicates are separately displayed in different color shades

Previous investigation of the phenomenon of strain degeneration in *T. reesei* was restricted to the Rut-C30 lineage. To find out whether the RL-P37 strain lineage is affected by this phenomenon and to what extent, the protocol for lab-scale ISD was applied for the *T. reesei* strains RL-P37 and GEN-3A, as well as the wild-type QM6a and Rut-C30 as reference strains. All were subjected to two rounds of the ISD with and without adding 20 mM DTT. The addition of DTT is reported to increase stress in the endoplasmic reticulum [26–28], and its addition was needed to degenerate QM6a, which is barely prone to degeneration [8]. For this reason, it was also included in this experiment, although the DTT condition is not relevant for industrial cellulase production. The obtained degeneration rates are provided in Fig. 2.

**Fig. 3:**
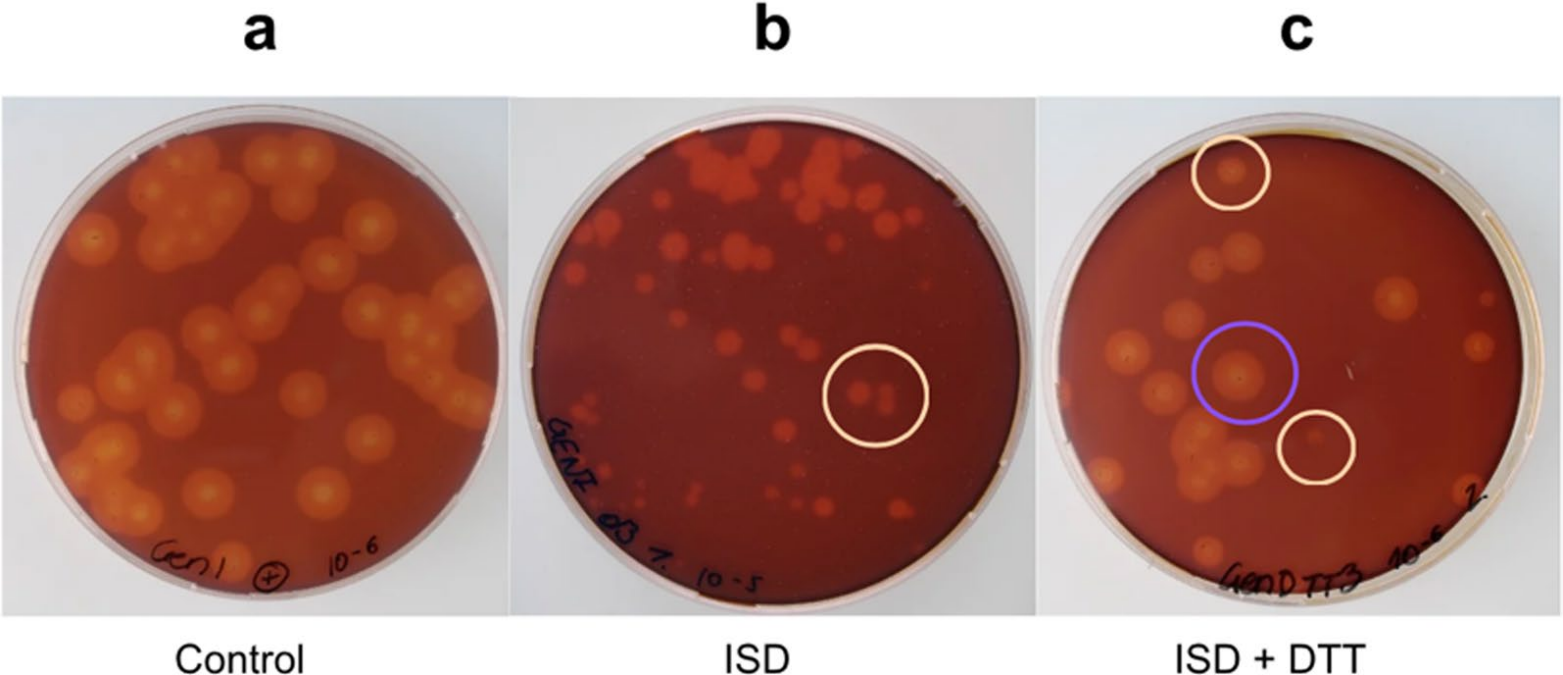
CMC agar plate screening of *T. reesei* GEN-3A before (**a**) and after the induced strain degeneration (ISD) protocol without (**b**) or with (**c**) the addition of 20 mM dithiothreitol (DTT). Examples of arising (cel-) colonies are marked in yellow and (cel +) in purple

After one round of the ISD protocol, the degeneration rate of RL-P37 increased from 5 to 11% (Fig. 2c), and for GEN-3A from around 10 to 50% (Fig. 2d). This gives evidence that degeneration occurs in this strain lineage and that GEN-3A, the more productive strain, is affected more strongly. Exemplary images of the CMC agar plate screening, from which the degeneration rate is calculated, can be found in Fig. 3.

The effect of DTT on degeneration varies between the strains. For Rut-C30 and GEN-3A, adding DTT results in a lower degeneration rate in the first round compared to the degeneration protocol without DTT. However, there is no difference anymore for the second round. In contrast, RL-P37 shows higher degeneration rates for both rounds with DTT than without. In this case, the degeneration rate increased from around 5% to 25% on average, but is still lower than for GEN-3A. Rut-C30 in this experiment had quite a high degeneration rate already before the degeneration protocol (around 25%), but increased to around 35% in the second round of degeneration (Fig. 2b). Only a minor increase in degeneration was found for QM6a (Fig. 2a), which is in accordance with the previous findings [8].

These findings highlight the complex and strain-dependent effect of DTT on degeneration. The initial reduction in degeneration rates for Rut-C30 and GEN-3A suggests that DTT may temporarily help cells cope with oxidative stress. DTT was shown to maintain intracellular redox balance by neutralizing free radicals and supporting antioxidant enzyme activity, thus protecting against oxidative damage [29]. However, the loss of this effect in the second round may result from prolonged exposure to DTT inducing ER stress, or from an adaptive response where its protective benefits are eventually outweighed by accumulating cellular damage. The contrasting response of RL-P37, which exhibited increased degeneration in the presence of DTT, indicates a higher sensitivity to ER stress by DTT in this strain.

**Fig. 4:**
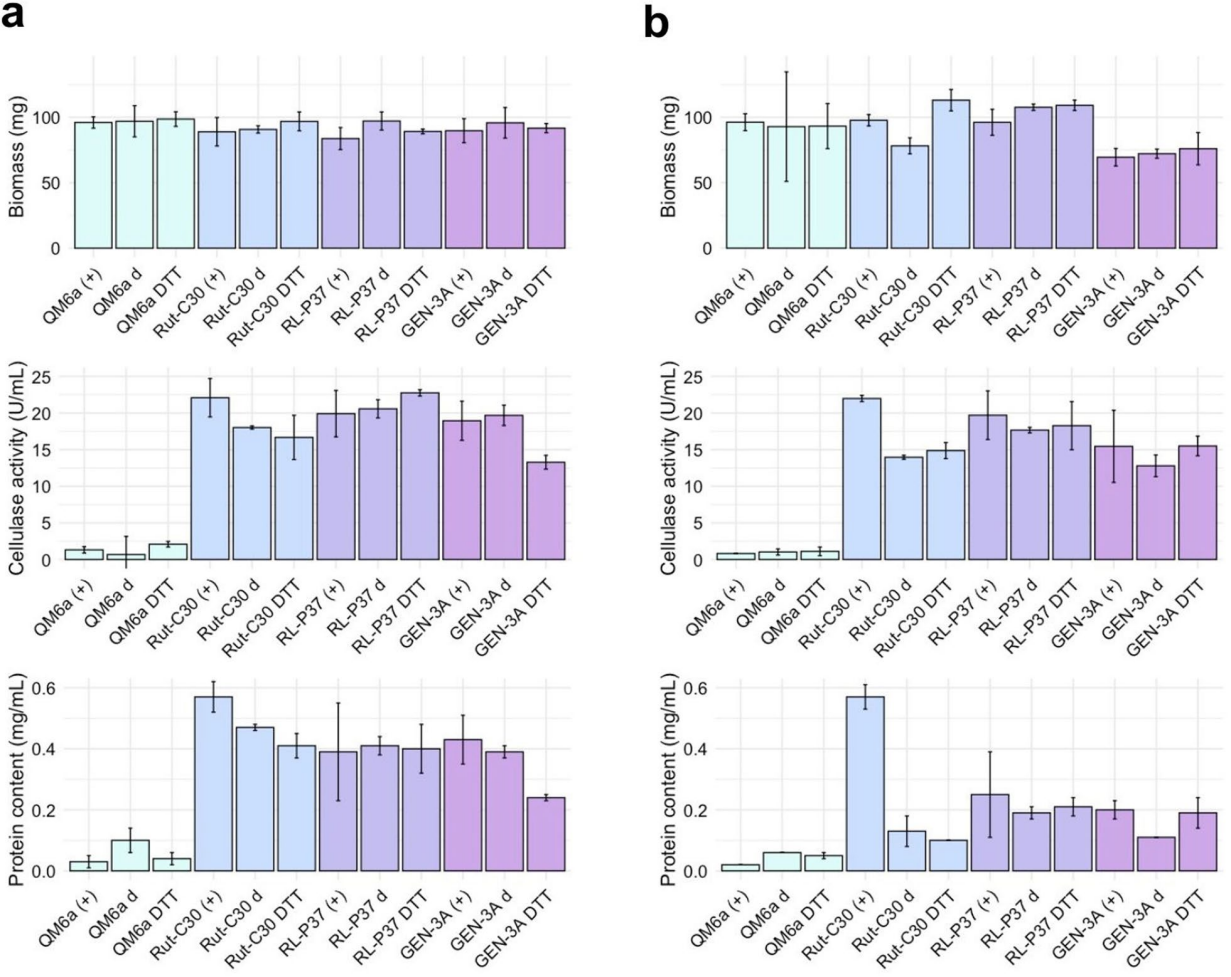
Phenotypic characterization of *T. reesei* strains QM6a, Rut-C30, RL-P37, and GEN-3A after one (**a**) and two (**b**) rounds of the ISD protocol. Biomass, cellulase activity in the supernatant, and total protein in the supernatant before the ISD protocol as positive control (( +)) and after the ISD protocol, either without (**d**) or with the addition of 20 mM DTT (DTT), were determined. Values are given as means from three biological and technical duplicates (cellulase activity) or technical triplicates (total protein), and bars are color-coded by strain

The phenotype, i.e., cellulase activity, total protein, and biomass, of all four strains before and after subjecting them to the ISD protocol was characterized, and the results are provided in Fig. 4.

An essential finding of this experiment was that cellulase activity and protein content were much lower than expected in the most productive strain, GEN-3A under these conditions (see Fig. 4, values for GEN-3A ( +)). The most likely reason for this is that the medium used in the ISD protocol was initially used for strains of the Rut-C30 lineage, which performed best (see Fig. 4, values for Rut-C30 ( +)), and does not seem to be optimal for cellulase production in the RL-P37 lineage. Therefore, the effects observed in this experiment for GEN-3A and RL-P37 may result from either strain degeneration or suboptimal medium conditions. As expected, Rut-C30 had reduced cellulase activity and protein content after being subjected to the ISD protocol (see Fig. 4, values for Rut-C30 d and Rut-C30 DTT), which is in accordance with the previous findings [8]. Also, the slight increase in biomass of Rut-C30 can be attributed to the rise of a non-producing subpopulation. The non-producers have a selective growth advantage, as they are not subjected to the high metabolic demands, which allows them to proliferate and potentially outcompete the original producing population [10, 30–32].

### Modification of the ISD protocol for the RL-P37 lineage of T. reesei

**Fig. 5:**
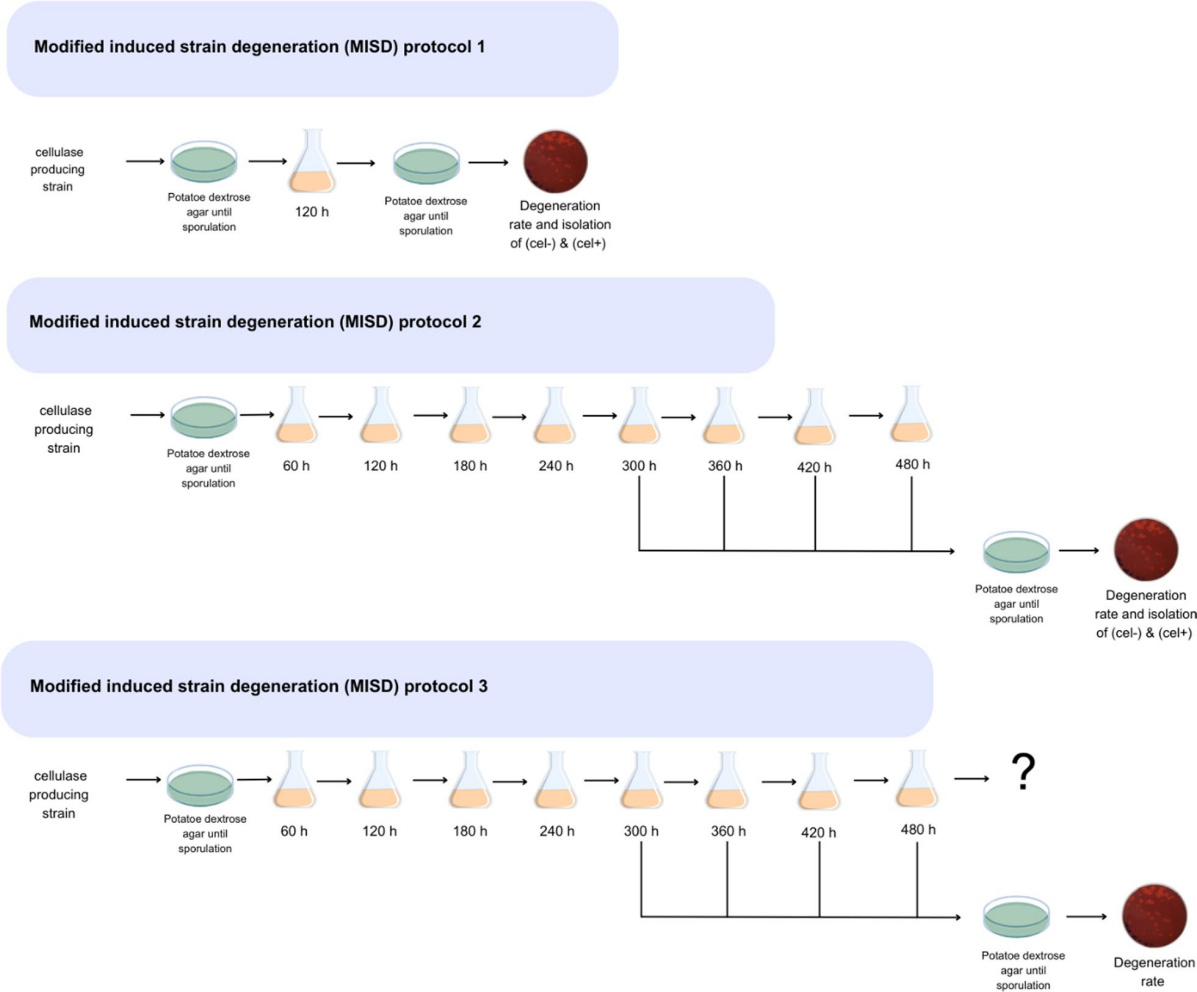
Schematic presentation of the three modified ISD (MISD) protocols

As a consequence of the obtained result described above, different modifications of the ISD protocol for the RL-P37 lineage were tested for RL-P37 and GEN-3A (Fig. 5).

**Fig. 6:**
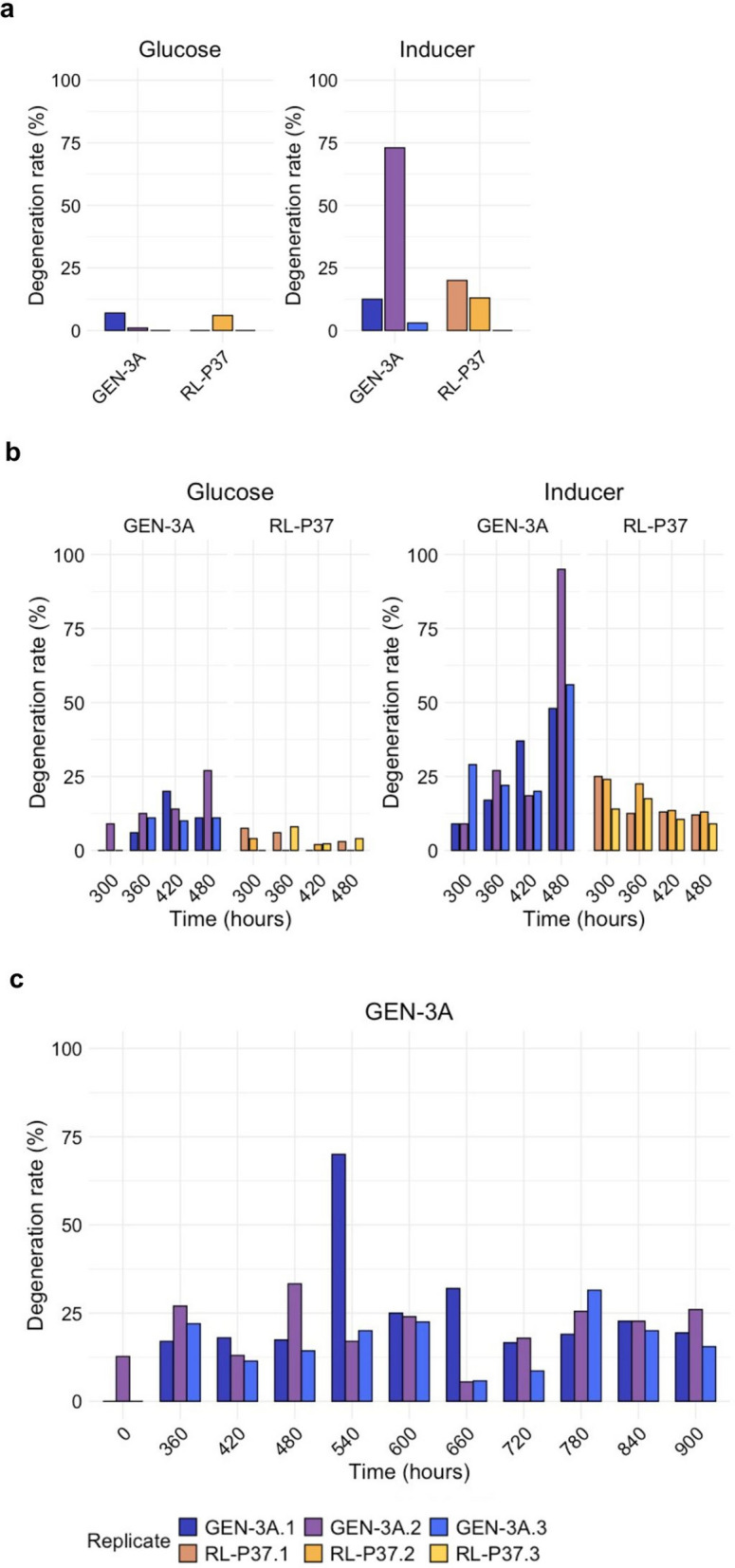
Degeneration rates obtained by the three MISD protocols. *T. reesei* strain RL-P37 (yellow shades) and GEN-3A (blue shades) were subjected to the MISD protocol 1 (**a**), protocol 2 (**b**), and protocol 3 (**c**) using cellulase production medium (Inducer) or minimal medium with glucose (Glucose). The three biological replicates are provided separately in different color shades

The first modified ISD (MISD) protocol is essentially the same as the protocol developed for the Rut-C30 lineage, with the difference that the cultivation medium was changed to the cellulase production medium provided by IFF. As a non-inducing control condition, cultivation was also performed on minimal medium with glucose. The second protocol has an extended cultivation of 480 h with a serial transfer of the fungal mycelia to fresh medium every 60 h to achieve a similar generation number as in the industrial production process, and at the same time to avoid that the pH of the medium drops below 3. The latter would affect cellulase expression and might lead to the observation of a different effect than the strain degeneration. The third protocol is the same as the second protocol; however, it was performed open-ended to determine whether the degeneration rates will further increase or stagnate. For protocols 2 and 3, mycelium samples were taken from cultures after 300 h growth every 60 h to determine the degeneration rates over time. In addition, (cel-) and (cel +) strains were isolated for further characterization at the end of protocols 1 and 2. The obtained degeneration rates are provided in Fig. 6.

Protocol 2 at 480 h resulted in an average degeneration rate of 66% in the case of GEN-3A and 12% for RL-P37 (Fig. 6b), resulting in higher degeneration rates than in protocol 1 (lasting 120 h) (Fig. 6a), with 29% and 11%, respectively. Monitoring the degeneration rate during protocol 3 led to the decision to end after 900 h because no drastic changes in the degeneration rate were observed between 600 and 900 h. Generally, the degeneration rate after 480 h did not increase significantly anymore, apart from one biological replicate at 540 h (Fig. 6c). Therefore, protocol 2 can be considered a successful modification of the ISD protocol for the RL-P37 lineage.

Importantly, this experiment reveals that degeneration is happening in this strain lineage when cultivated under conditions that are close to the industrial cellulase production process. RL-P37 had a much lower degeneration rate than GEN-3A, thus, the severity of the phenomenon is also related to the productivity of the strain, which is comparable to the findings of the Rut-C30 lineage [8].

### *Phenotype of isolated (cel-) and (cel* +*) strains*

**Fig. 7:**
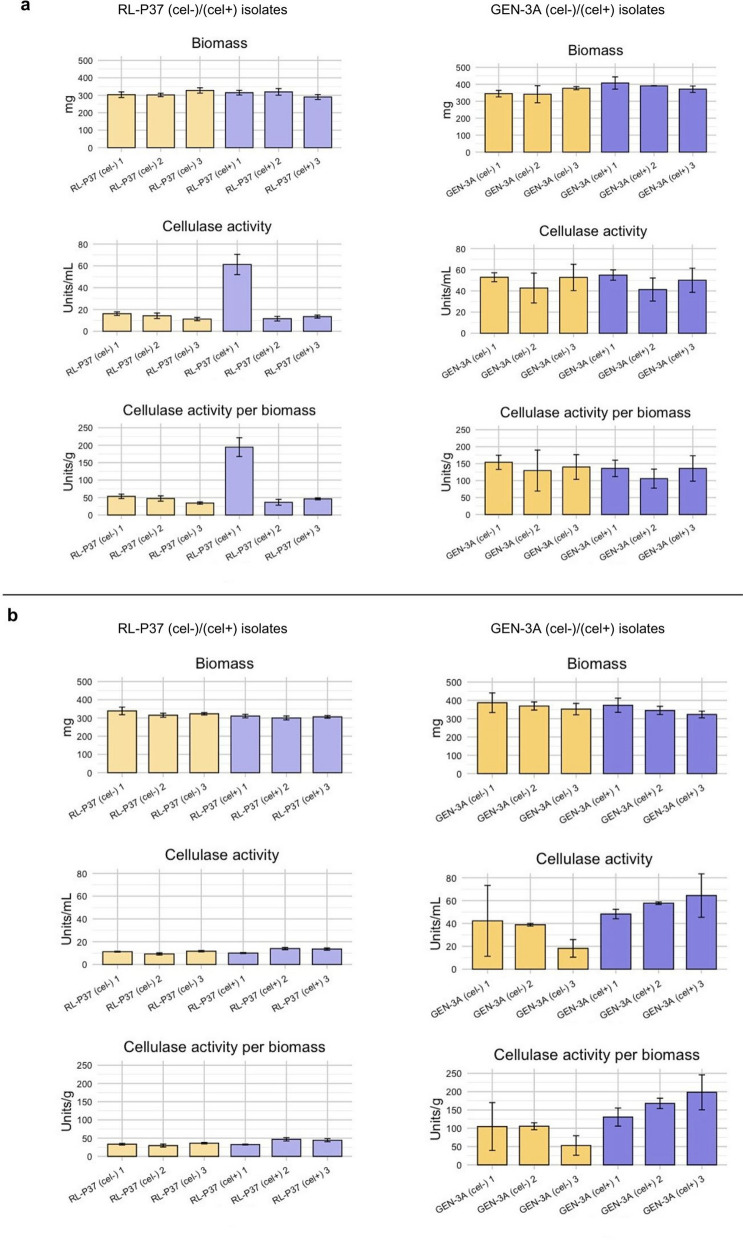
Phenotype of isolated (cel-) and (cel +) strains. Three (cel-) (yellow bars) and (cel +) (purple bars) strains (1, 2, 3) from RL-P37 and GEN-3A isolated after the MISD protocol 1 (**a**) and 2 (**b**) were grown under cellulase-inducing conditions, and cellulase activity in supernatants and dry biomass was determined. Values are means of biological triplicates (RL-P37) or duplicates (GEN-3A) and technical duplicates, with error bars providing the standard deviation

The (cel–) and (cel +) strains isolated from MISD protocols 1 and 2 were cultivated in cellulase-inducing conditions to compare cellulase activity and growth, verify their supposed phenotype, and select strains for further experiments. In accordance with the obtained degeneration rates (Fig. 6), the strains isolated after protocol 2 are closer to the expected phenotype than the isolates after protocol 1 (Fig. 7, compare a and b). Especially in the case of GEN-3A, the (cel–) strains isolated after protocol 2 consistently have a decreased cellulase activity compared to the (cel +) strains, while there is no apparent difference for the isolates from protocol 1. On average, the cellulase activity for the three GEN-3A (cel–) strains is 33 Units/mL and 57 Units/mL for the three GEN-3A (cel +) strains, and thus, the (cel–) strains show a 42% reduction in cellulase activity compared to the (cel +) strains. This depicts an essential decrease in cellulase activity that could lead to substantial economic losses in production. The percentual activity reduction between the most pronounced phenotypes, i.e., GEN-3A (cel–) isolate no. 3 with only 18 Units/mL and (cel +) isolate no. 3 with 64 Units/mL, is 72%. In the case of RL-P37, there was not such a substantial difference between the (cel–) and (cel +) isolates found compared to GEN-3A, but they follow the same pattern, apart from one (cel +) strain isolated in protocol 1 that shows much higher activity than all others. This enforces that the degeneration protocol 2 is suitable for the degeneration of the RL-P37 lineage, and the degeneration effect is much more pronounced for GEN-3A. Also, it was confirmed that the (cel–) and (cel +) isolates show the expected phenotype and are helpful for further experiments.

### Stability of the cellulase-expressing phenotype

**Fig. 8:**
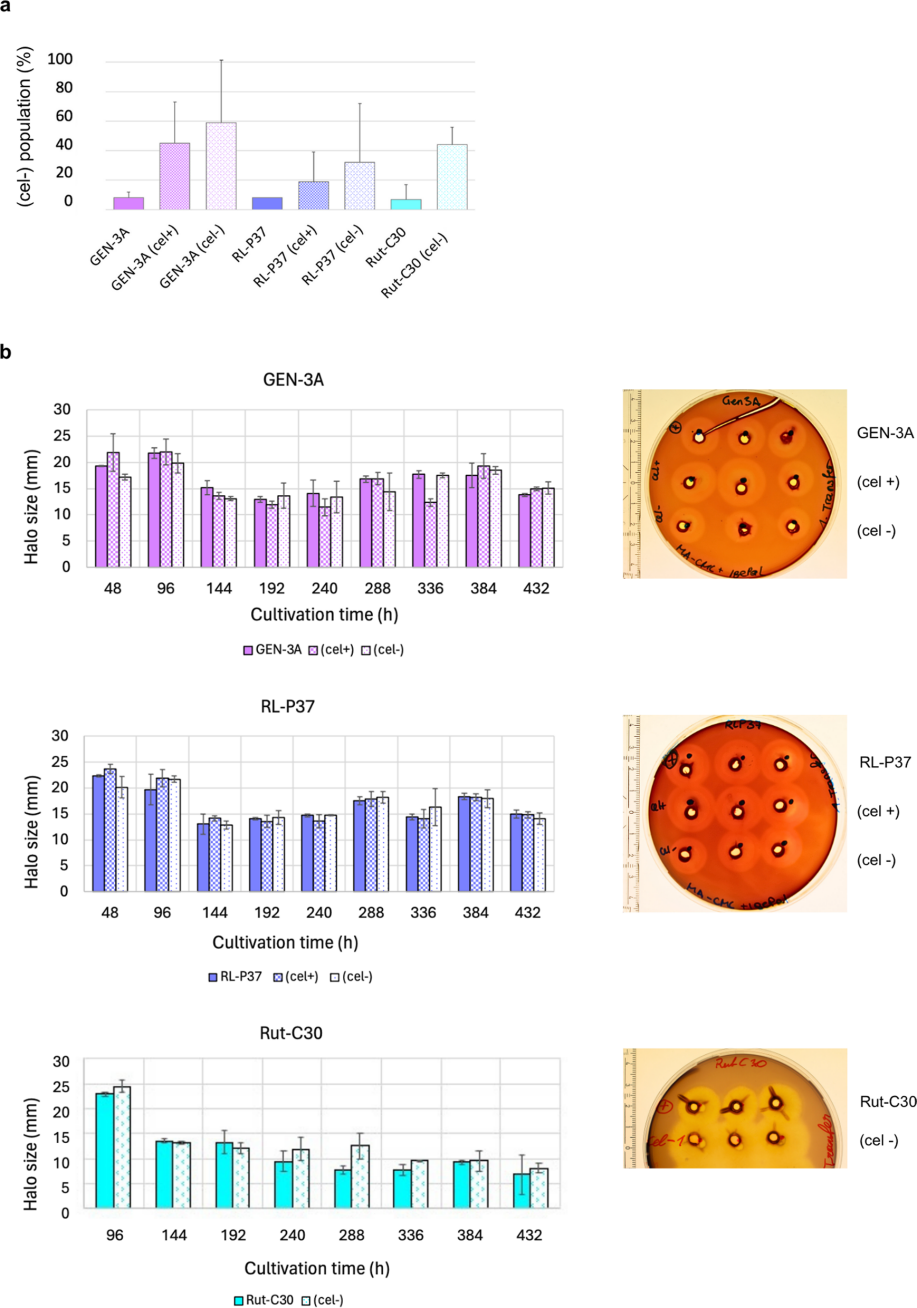
Monitoring the cellulase-expressing phenotype. **a** Degeneration rates of GEN-3A, RL-P37, Rut-C30, and their respective (cel-) and (cel +) isolates. The values are means of biological duplicates. **b** Halo sizes of RL-P37, GEN-3A, Rut-C30, and their respective (cel-) and (cel +) isolates on CMC agar plates during serial sub-cultivation. Values are means of biological triplicates with error bars indicating the standard deviation

We performed two subsequent experiments to clarify whether the observed (cel-) or (cel +) phenotype is maintained over multiple generations or potentially reversed. First, it was necessary to understand how homogenous an initial spore population is regarding its cellulase-producing phenotype. For this, diluted spore solutions of the strains RL-P37, GEN-3A, and their respective (cel-) and (cel +) isolates were plated on CMC agar plates to determine the initial degeneration rate. Rut-C30 and two Rut-C30 (cel-) strains were used as controls. One could have expected that the parent and the (cel +) strain only show a minor (cel-) percentage, and the (cel-) strain primarily has (cel-) colonies. Notably, we found that none of the strains has a 100% homogeneity (Fig. 8a). While the parent strains indeed had a low degenerated population, both the (cel-) and (cel +) strains had a much higher degenerated population, but (cel-) strains did also not exclusively show (cel-) colonies. These results are similar for all three strains.

The second experiment tested the stability of the cellulase-producing phenotype of the above-mentioned strains by serial sub-cultivation in cellulase-inducing conditions. For this, overgrown agar plugs of the same size were transferred every 48 h on fresh CMC agar plates. The halo size was recorded over a total time of 432 h and can be considered as an indicator for cellulase expression. For RL-P37 and GEN-3A, the first time point nicely confirmed the expected phenotypes, as the halo diameter of the (cel-) strain was the smallest, followed by the parent strain, and the (cel +) strain yielding the most enormous colony size (Fig. 8b). The Rut-C30 (cel-) strain had a bigger halo size than its parent at the first time point (Fig. 8b). Interestingly, over time, all strains—regardless of whether parent, (cel-) or (cel +) strain—lost cellulase expression, reflected by a decrease in halo size compared to the early time points (Fig. 8b). These results demonstrate a degeneration trend for all strains and that the (cel-) and (cel +) phenotypes are initially distinct, but when further cultivated under cellulase-inducing conditions, the phenotypes gradually converge.

Together, these findings indicate that strain degeneration is not a transient or reversible phenotypic switch, but rather a progressive and persistent process maintained across generations. The heterogeneity observed in spore populations further supports this. Rather than being uniform, both (cel −) and (cel +) isolates contain subpopulations with differing cellulase production capacities. While parent strains predominantly produce (cel +) colonies with only a small proportion of (cel −) cells, derived isolates—both (cel −) and (cel +)—show an increase in (cel −) colony formation. The presence of (cel −) cells even within (cel +) isolates again suggests that degeneration is a gradual process in which (cel −) cells progressively gain a selective advantage. These results support a model in which degeneration is driven by stable, potentially irreversible genetic or epigenetic changes, leading to a shift on the population level rather than a simple on/off phenotypic switch. From an industrial perspective, this progressive loss of productivity makes it necessary to maintain ongoing monitoring and strain quality control throughout the production process.

### *Morphology of (cel-) and (cel* +*) strains*

**Fig. 9.**
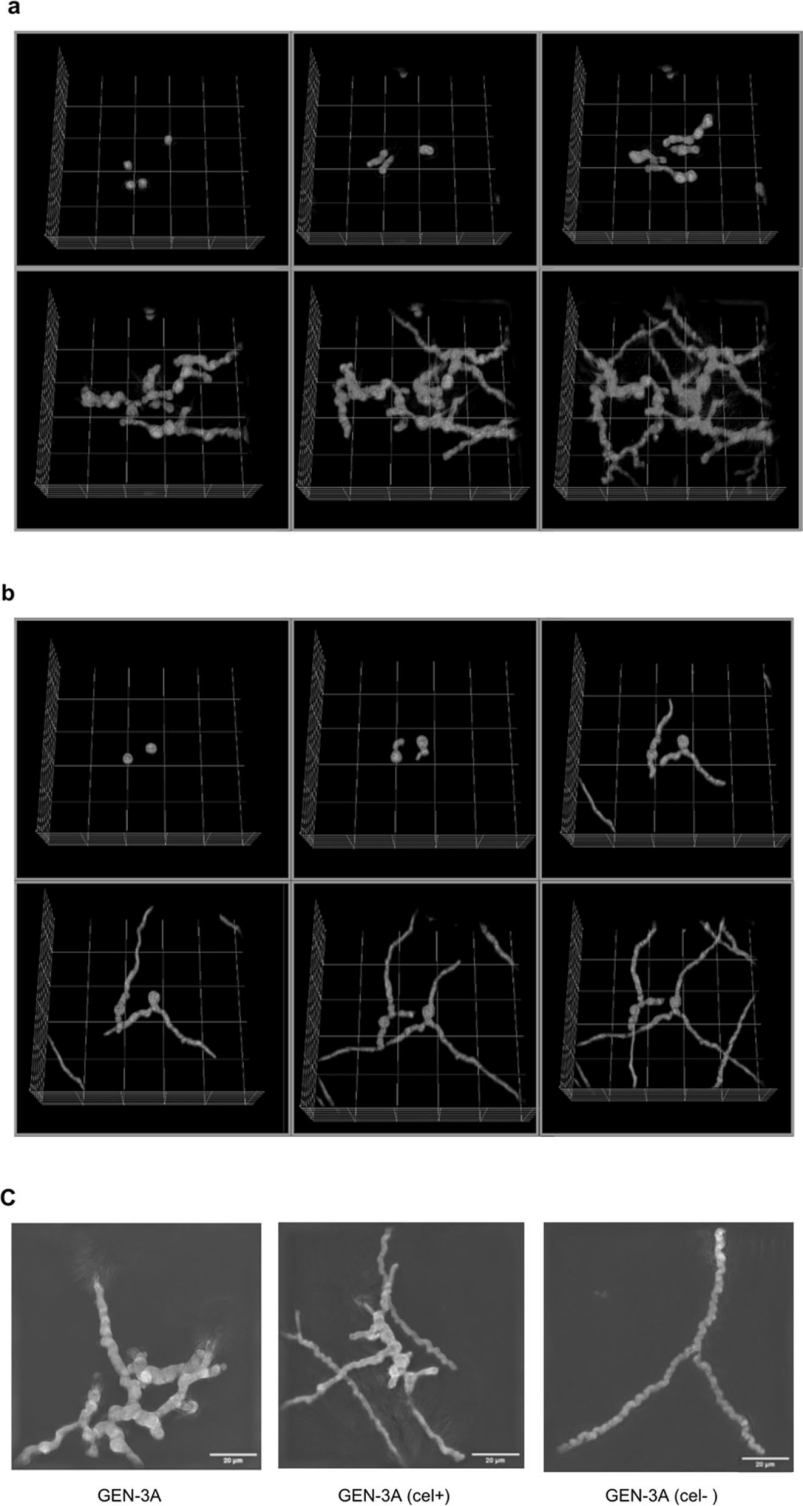
3D-rendered image sequence of GEN-3A cultivated in cellulase-inducing conditions (**a**) or on glucose (**b**). The images were taken with the Nanolive Cell Explorer over a time period of 18 h. The top left image shows spores, the top middle image shows the start of spore germination at around 2 h, and the top right to bottom right image shows hyphal growth from around 6 to 18 h. **c** 3D-images of GEN-3A and GEN-3A (cel-) no. 3 and (cel +) no. 3 after 18 h of cultivation in cellulase-inducing conditions using the single-shot function of the Nanolive Cell Explorer. The scale bar is set to 20 μm

Cellulase productivity of *T. reesei* is suspected to be linked to its morphology, and it was already reported that a degenerated population from strains in the Rut-C30 lineage exhibits a different morphology [8]. Therefore, we investigated whether a change in morphology accompanies the degeneration phenomenon of the RL-P37 lineage and how it is characterized. For this purpose, we observed colony and hyphal growth of the (cel-)/(cel +) strains compared to the parent strain. The colony morphology was observed by reflected light microscopy on agar plates with minimal medium and CMC or glucose as a carbon source. However, no significant difference was visible between the different strains. Hyphal morphology was observed first with a light microscope and later using the Nanolive Cell Explorer, which uses a holotomographic imaging method to create 3-dimensional images and videos. One crucial observation was that the morphology of GEN-3A already differs significantly depending on the carbon source. In the induced condition, the hyphae are swollen/bulbous, and there is a lot of branching (Fig. 9a), while in the non-induced condition, the hyphae are much thinner and longer, and there is less branching (Fig. 9b). In contrast, no significant difference exists between induced and non-induced conditions for RL-P37 (data not shown).

This finding suggests that the bulbous, swollen hyphae are important for the cellulase hyperproduction, and this is in accordance with other literature reports: swollen, shorter, and highly branched hyphae were associated with cellulase hyperproduction in *T. reesei* strain PC-3–7 [33]. In *T. reesei* Rut-C30, this morphology was dependent on the cultivation with Avicel as an inducing compound [34]. Compared to Rut-C30, the cellulase hyperproducing mutant DES-15 also showed shorter, more bulbous, highly branched hyphae [34]. Further, this morphology, due to an improved rheology, was used for a screening attempt to improve protein production with *T. reesei* strains in a fed-batch process [35].

The most substantial difference in morphology of the GEN-3A (cel-) and (cel +) strains is visible in cellulase-inducing conditions. It was found that the (cel-) strains show thinner hyphae with fewer bulbs, and the (cel +) strain exhibits a morphological mixture between the parent and the (cel-) strain, as both bulbous and very thin hyphae are visible (see Fig. 9c). The (cel-)/(cel +) strains show a similar morphology as the parent strain on glucose. Hence, one could conclude that the inducing effect is not visible anymore in the degenerated phenotype. Interestingly, for RL-P37, the difference in morphology between the isolates compared to the parent and inducing versus non-inducing conditions is not so substantial. This aligns with the fact that it generally produces fewer cellulases and degenerates less.

Together, these results indicate that there is a distinct morphology associated with the cellulase-expressing phenotype and also with the degeneration phenomenon of hyperproducing strains, which might be helpful for future screening purposes of the degenerated population and also help to better understand the importance of morphology for cellulase hyperproduction.

## Conclusion

This study provides the first characterization of strain degeneration in the RL-P37 lineage of *T. reesei*, advancing previous observations restricted to the Rut-C30 lineage. Our findings reveal that degeneration is not restricted to Rut-C30 and derivatives, but also affects RL-P37 and its hyperproducing descendant GEN-3A. A clear correlation between higher cellulase productivity and increased degeneration susceptibility was identified. Degeneration was linked to a reduction in cellulase activity and distinctive morphological changes, in particular, the loss of bulbous, highly branched hyphae linked to the productive phenotype. The identification of a potential morphological marker in degenerated strains would provide a promising basis for early detection and screening strategies. However, at this point, this is merely an auxiliary observation and requires further investigation to verify causality. Furthermore, our observations indicate that degeneration—regardless of the strain lineage– is rather progressive and maintained over multiple generations, posing a serious challenge for industrial enzyme production. This insight strongly suggests elucidating the molecular mechanisms causing the strain degeneration to eventually identify strategies to prevent it.

## Data Availability

All data generated or analyzed during this study are included in this published article and its supplementary information files.

## Abbreviations

CMC: Carboxymethylcellulose
DTT: Dithiothreitol
ER: Endoplasmic reticulum
GEN-3A: Hypercellulolytic descendant strain of *Trichoderma reesei* RL-P37
ISD: Induced strain degeneration
MA medium: Mandels–Andreotti medium
MISD: Modified induced strain degeneration
PDA: Potato dextrose agar
(cel −): Degenerated subpopulation of *Trichoderma reesei* with low or no cellulase production
(cel +): Still cellulase-producing subpopulation of *Trichoderma reesei*
QM6a: Wild-type *Trichoderma reesei* strain
RL-P37: Industrial *Trichoderma reesei* strain derived from NG14
Rut-C30: Hypercellulase-producing *Trichoderma reesei* strain derived from NG14
*T. reesei*: *Trichoderma reesei*
